# Factors Influencing Undernutrition among Female Adolescent Students in Darchula District, Nepal

**DOI:** 10.3390/nu15071699

**Published:** 2023-03-30

**Authors:** Deepika Giri, Nitaya Vajanapoom, Uma Langkulsen

**Affiliations:** Faculty of Public Health, Thammasat University, Bangkok 12120, Pathum Thani, Thailand

**Keywords:** undernutrition, late adolescent, intergenerational cycle of malnutrition, dietary diversity, food security, Nepal

## Abstract

Failure to understand and address the problem of malnutrition and its associated factors among female adolescents could lead to a vicious cycle of intergenerational malnutrition. A cross-sectional study was conducted in six secondary schools of four rural municipalities in Darchula District, Nepal. Data collection for the study was conducted from November 2021 to February 2022. Four hundred female adolescent students aged 15 to 19 years old were selected using quota sampling. The study aims to examine the prevalence of malnutrition by assessing different levels of body mass index (BMI) that is thinness (BMI less than 18.5 kg/m^2^), normal (18.5 kg/m^2^ to 24.9 kg/m^2^), overweight (25 kg/m^2^ to 29.9 kg/m^2^) and obese (BMI more than or equal to 30 kg/m^2^). Along with BMI, factors associated with undernutrition, here identified as thinness, are assessed using a structured questionnaire. Additionally, key informant interviews and review of interventions was performed to understand the gaps in nutrition-related policies and programs of school going adolescents in the study district. Quantitative data analysis included a prevalence study and chi-square test along with simple and multiple logistic regression to obtain crude and adjusted odds ratio at 95% confidence interval for the significant factors with *p* < 0.05 identified in the chi-square test. Thematic analysis and reviews were used for the synthesis of qualitative data. The results show a 24.7% prevalence of thinness in the study participants. Pre-mensuration status (OR = 5.015, CI = 1.257–20.011, *p* < 0.022), father having a monthly paying job (OR = 4.384, CI = 1.135–16.928, *p* < 0.032), father’s foreign employment (OR = 6.96, CI = 1.649–29.377, *p* < 0.008), household (HH) food insecurity status (OR = 2.079, CI = 1.182–3.658, *p* < 0.011) and grain/roots/tuber as most commonly bought food (OR = 9.487, CI = 1.182–76.138, *p* < 0.034) were found to be significantly associated with thinness. Information from the qualitative part identified gaps in existing interventions for an improved nutritional outcome among school going adolescent females. Further studies to understand the nutritional practices and its contributory factors in relation to thinness is encouraged. Stakeholders are urged to effectively address the shortcomings in existing interventions and adopt a more adolescent-centered approach to enhance the nutritional status of female adolescents.

## 1. Introduction

Whereas nutritional well-being is imperative for an individual to reach their full mental, physical, social and economic potential, malnutrition can have serious negative consequences for an individual. Malnutrition can be in various forms, of which undernutrition is one, and can further be classified as stunting (low height-for-age), wasting or thinness (low weight-for-height), underweight (low-weight-for-age, used for children) and micronutrient deficiencies [1]. Although many developing nations are struggling with the challenges posed by undernutrition and developed countries with overnutrition, there are also numerous countries, irrespective of their development status, that are facing a double burden of malnutrition. South Asia faces predominantly a very high prevalence of thinness (10.9%) compared with any other region of the world, with a very low average annual reduction of 1.1% [2].

Globally, people in all phases of life face some threats of enduring different nutrition-related problems. However, nutrition during adolescence is not only expected to meet the existing nutritional needs but is also crucial to counterbalance childhood malnutrition complications if there are any and readies the body to enter adulthood [3]. During this phase, approximately 15–20% of height, more than half of skeletal mass and half of adult bodyweight is attained. Therefore, being unable to meet an adolescent’s high nutritional demand can make them more vulnerable to undernutrition [4,5]. Along with numerous factors at the individual and community levels, the intricate socio-economic structure, child marriage, early pregnancy and childbirth additionally contribute to undernutrition among female adolescents in developing countries. Worldwide, 21% of late adolescent girls (15 to 19 years old) are married, whereas the number goes as high as 30% in the least developed countries. A total of 21 million adolescent girls in this age group are pregnant in low- and middle-income countries (LMICs) [2,6,7]. During pregnancy, the adolescent body, which is already in ample need of nutrients, is challenged to sustain the nutrient requirement for foetus as well, sometimes compromising the growth and development of either one or both. Undernourished adolescents could initiate an intergenerational cycle of malnutrition as they have higher chances of giving birth to children with health and nutrition complications such as cognitive impairments, low birth weight (LBW), small for gestational age (SGA), preterm birth, poor bone development and lower resistance to infections along with a higher risk of disease and death throughout their lives [8,9,10,11].

During the unique phase of late adolescence various individual-, household (HH)-, community- and environment-related determinants contribute to shaping nutritional well-being [12,13]. Among numerous factors, inadequate dietary intake, food diversity, amount of food consumption and skipping of meals are found to directly affect body mass index (BMI) [14,15,16,17,18]. Similarly, a few studies have suggested a direct association of diseases such as malaria and worm infestation, menarche and status of mensuration cycle (as it can interfere with the absorption of nutrients) can cause requirements for extra nutrients to recover or can also cause nutrient wastage [16,19,20,21]. Along with these direct cause factors, there are various other underlying factors. One of these is inadequate availability and access to nutritious food on own farm, market, community and HH levels [22,23,24,25]. Culture is also one of the underlying factors and can influence food taboos such as restrictions of certain foods during mensuration, decision makers of the family, what is grown in the field, expenditure on food, intrahousehold food distribution, gender inequalities in food distribution and inequalities during seasonal food deficit [22,23,26,27]. Sociodemographic status such as income, size of the family, religion, ethnicity, education and occupation of parents singularly or jointly interplays with other determinants and can influence decision making in the family, the purchasing power of a family, consumption of nutritious and quality food and food distribution and hence affect the nutrition status of family members [5,16,17,18,28]. Additionally, hygiene and sanitation in relation to persons, food, water and environment are also linked with infectious diseases and hence contribute to undernutrition and repeated infections [16,24]. Along with these factors, an adolescent’s problem of undernutrition is also influenced by their knowledge, attitude and behaviour towards nutrition and its related factors [29,30].

Around 1.4 million people (11% of the total female population) in Nepal are adolescent girls [31]. In Nepal, among females aged 15 to 19 years, 30% are thin (BMI less than 18.5 kg/m^2^), whereas 13% of other women of reproductive age are thin [32]. Similar to other LMICs, in Nepal females in their late adolescence have a high prevalence of early marriage, pregnancy and childbearing that is more prominent in rural areas. The intergenerational effect of malnutrition is also evident as the prevalence of LBW, birth defects and the mortality rates of neonates, infants and children under 5 years of age are considerably higher among children of young as well as thin mothers (lower BMI) [8,32,33,34,35]. Despite this, very little is known about the problem of undernutrition among late adolescent females and its associated factors in various regions of the country, in particular, the Darchula district. From national statistics, 10% of the total population are females in their late adolescence and there is a high prevalence of child marriage [31]. The overall development indicators such as human development index (HDI), gender empowerment measure (GEM) and human poverty index (HPI) in the region including the Darchula district are not satisfactory [36]. These might have contributed to the poor development, gender disparities and poor performance in education, health and nutrition of female adolescents in these regions.

Various policy, commitments, policy reviews and guidelines have prioritized key areas to strengthen efforts to address the problem of malnutrition among adolescents. Adolescents, particularly in their later phase (15 to 19 years), are unique in terms of their physical growth spurt and emotional, mental, social and behavioural development. Furthermore, with the increasing number of adolescent girls at school, it is becoming harder to implement and benefit from only community-based interventions [37]. Additionally, adolescents are mostly incorporated in maternal and child focused programs, creating the possibility of underestimating their needs and failure to improve their nutritional statuses. Along with programmatic lag, the existing gap in the research for the understanding of the issue of adolescent undernutrition, along with implementation of evidence-based multi-sectorial and innovative ideas to address the problem is identified [38,39].

This school-based study aimed to investigate the prevalence of malnutrition in terms of BMI and factors influencing low BMI or thinness among female adolescent students in the Darchula district of Nepal. The study further explored the existing policies and interventions for gaps in addressing the issue. Understanding the magnitude of the problem in a rural setting of an LMIC and factors associated with it will be significant for planning of targeted nutritional policies and programs considering the importance and need of adolescent-focused interventions in future. This would add to the existing body of knowledge on the topic and encourage more studies on adolescent undernutrition.

## 2. Materials and Methods

### 2.1. Study Site

Nepal has 77 districts, 3 different ecological zones and 7 provinces. Of these, the study was carried out in one district of a Himalayan region in province 7. The study intended to focus on the seven rural municipalities in the setting of Darchula District, Nepal, consisting of Malikaarjun, Lekam, Byans, Duhun, Apihimal, Naugadh and Marma. However, not all of these areas were accessible due to unexpected constraints during the data collection amidst the coronavirus disease (COVID-19) pandemic. Therefore, the study was confined to the accessible rural municipalities of Malikaarjun, Lekam, Byans and Duhun.

### 2.2. Study Design

A cross-sectional study was conducted among female students aged 15–19 years enrolled in classes 9–12 of schools in the four rural municipalities of the Darchula district. The study used a mixed method, where the quantitative part attempted to assess the prevalence of malnutrition in terms of the BMI of the students and the factors associated with their thinness, whereas the qualitative part explored the gaps in policies and interventions addressing adolescent nutrition in the schools.

### 2.3. Study Population

The study population were female students aged 15 to 19 years enrolled in any class from 9 to 12. Participants were recruited from two secondary and four higher secondary schools. A prior meeting was conducted with school administration for approval of data collection and at the same time basic information on the females enrolled in classes 9–12 was gathered. During the 1st interaction with students and prior to interview, students of age 15 to 19 years were identified based on the age self-reported by students. Among the late adolescent female students in the rural schools of the four rural municipalities, they were excluded from the study if they had the following conditions on the day of data collection: not being present in the class, being pregnant, having a disability with limited physical movement or having a chronic illness, i.e., diabetes, allergy, asthma or cardiovascular diseases. In addition to this, the school’s prior approval for the selection of classes was sought in such a way that their lessons were not unduly interrupted. Eligible key informants for qualitative data collection were the chairperson or health focal person of the ward office and the principal or health focal person of the selected schools. Those available and interested in the interview were included in the study. A signed consent form was sought from all of the potential participants and the guardians of the students that were aged below 18 years old. Ethical approval for the study was obtained from the Human Research Ethics Committee of Thammasat University (Science) (HREC-TUSs) (COA No. 106/2563), Nepal Health Research Council (NHRC) and the Education Development and Coordination Unit (EDCU) of the study district.

### 2.4. Sample Size

For the assessment of the prevalence and the factors associated with thinness, a sample size of 383 was calculated based on the information from a study conducted in 13 districts of Nepal [18], i.e., the prevalence of thinness (75.9%) among adolescent students who did not have latrines at home (60.5%). The estimated sample size gives 80% power to estimate an odds ratio as low as 2.52 with a 5% significant level. To deal with possible non-response, invalid or incomplete response, and sampling error, the sample size was increased to 400 students. The sample size for key informant interview (KII) was 12 people (health focal person/principal/ward chairperson/health focal person) from the ward.

### 2.5. Sampling Technique

Quota sampling was used to select students from the Malikaarjun, Lekam, Duhun and Vyans rural municipalities. The number of students from each rural municipality was determined based on the proportion of female students enrolled in classes 9–12 in the secondary and higher secondary schools. The eligible students were conveniently selected from the largest school in each municipality until reaching the required number of students for that particular municipality. If necessary, the eligible students were selected from the second largest school and so on, until reaching the required number of students for each municipality. Using this procedure, 130 students from a school in Malikarjun, 121 from Lekam, 75 students from Duhun and 74 students from three schools in the Vyans rural municipality were included.

For the KII, 12 eligible key informants (6 principal/health focal persons from 6 selected schools and six officials from wards where the schools were located) were identified and selected. However, only nine agreed to participate; principals from three schools, health focal persons from two schools, chairpersons from three different wards and one health focal person from a ward office where the schools were located participated in the study.

### 2.6. Data Collection

Data collection from students and key informants for both quantitative and qualitative study began in November 2021 and ended in February 2022.

#### 2.6.1. Quantitative Data

The data on dependent and independent variables were collected using a structured questionnaire. Most of the questions were adapted from a previous nutritional survey conducted in 13 districts of Nepal [16]. Dietary diversity was assessed using Food and Agriculture Organization (FAO) guidelines. Household food insecurity access prevalence was identified using a tool developed by Food and Nutrition Technical Assistance (FANTA), questions on attitude were developed with reference to a Knowledge Attitude Practice (KAP) manual and questions for mensuration cycle was prepared based on reference from a previous study [40,41,42,43]. To fulfil the objective of the study, a few parts were modified for this study. Expert validation of data collection tools (questionnaire and interview guideline for KII) was performed by an external expert and the advisor for the study. A pre-test of the questionnaire was conducted among 40 female adolescents in a school from a rural area of the study district. Details of the dependent and independent variables are described below.

- BMI: The height and weight of the students were measured to calculate BMI (weight in kilogram divided by square of the height measured in meter). The height was measured using a stadiometer by making students to stand straight on a uniform base. To measure body weight, a weight scale laid on a uniform base where students were asked to step upon bare footed; extra weight due to outer heavy clothes, bags or other belongings were avoided. Organizations such as the World Health Organization (WHO) and Centers for Disease Control and Prevention (CDC) have provided different methods and cut-off points for identifying categories of BMI for people from different age groups and sometimes even for people from different regions. Here, the student’s BMI distributions were compared with a reference used by the Nepal Demographic and Health Survey (NDHS) for determining the BMI of women aged 15 to 49 years. All individuals with BMIs of less than 18.5 kg/m^2^ were classified as thin, BMIs of 18.5 kg/m^2^ to 24.9 kg/m^2^ were classified as normal, BMIs from 25 kg/m^2^ to 29.9 kg/m^2^ were classified as overweight and BMIs more than or equal to 30 kg/m^2^ were considered as obese [32].
- Socio-demographic characteristics: These included basic information about students such as age, marital status class, religion, ethnicity, education of students, education and occupation of parents, income, family size, head of HH, etc [18]. As the study was focused only on females, students who self-reported themselves as females were included.
- Culture: This is a vague term but here culture-related information was confined to: decision maker of family and food taboos in the form of food restriction during mensuration [22,26,27].
- Dietary Diversity: Diet diversity or dietary diversity was identified in terms of the number of food groups included in the diet within the last 24 h and consumption of five or more than five food groups was considered as diet diversity [41].
- Disease: For disease, the study was limited to only worm infestation and malaria [16,44].
- Mensuration cycle: This included status of menarche and mensuration cycle. The cycle was classified as normal or not based on repetition of the cycle, days of blood flow and volume of blood flow [43].
- Inadequate nutritious food: This was based on information on food availability at own farm, market, community or at own house, food stock, food purchase, basis of decision for selection of food and food allocation at intra-household level [22,24,25,45].
- Household food insecurity assessment: Data were collected to measure the prevalence of food insecurity at the HH level and the status within the past month. Prevalence was based on three domains: anxiety and uncertainty about HH, insufficient quality and insufficient food intake and its physical consequences. Four levels: food secure, mildly food insecure, moderately food insecure and severely food insecure were identified based on identification of the occurrence of problems with yes or no answers and follow-up questions related to the frequency of occurrences of specific problems [40].
- Nutrition related KAP: Knowledge on nutrition was assessed on the basis of multiple-choice questions related to source and importance, with each correct response equal to 1 score. The total score was divided into different percentages where: 0 to 25 percent of correct answers was considered poor; 26 to 50 was average; 51 to 75 was satisfactory and 76 to was a good level of knowledge. Attitude in this study was about how the students felt about nutrition-related recommendations, risk of malnutrition and how confident they were in adopting ideal nutrition. This section had three-point Likert scale; negative responses were coded 1, neutral or uncertain responses were coded 2 and positive responses were coded 3. The mean was calculated for the responses of questions in each of the above 4 categories of attitude-related assessment. If the mean was from 1 to 1.67, the attitude of students for the category was identified as negative, 1.68 to 2.33 indicated the attitude was neutral and from 2.34 to 3 the attitude was indicated as positive [42]. Nutrition practice: This is related to the day-to-day nutritional habits of students such as type of staple food, consumption patterns of nutritious food and junk food, skipping of meals, meals per day, food consumed during school breaks and habits of smoking and drinking.
- Hygiene and sanitation: Hygiene refers to personal hygiene, related to hand washing and wearing shoes, whereas sanitation was about drinking water, its purification and having latrines at home [18].

#### 2.6.2. Qualitative Data

National nutrition policy and school health nutrition policy of Nepal was reviewed to understand the existing interventions in addressing the issue of malnutrition among school-going female adolescents and possible barriers. Guidelines for programming and implementation of effective adolescent nutrition interventions, commitments at international level, key nutrition-related movements and policy recommendations were also considered. The review was performed by the principal investigator. This was followed with in-depth interviews with key informants from school and ward offices using an interview guide. Along with the guideline, there were open-ended probing questions to ensure having sufficient information regarding the topic. Interviews were conducted in person and continued for 60 to 90 min. An interviewer that was well oriented about the study, ethical considerations, quantitative and qualitative methods and data collection processes was involved. Prior to the actual interview, repeated mock interviews were conducted with the support of the principal investigator. While quantitative data collection from students was carried out, simultaneously document reviews and interviews for qualitative data were also conducted.

### 2.7. Data Analysis

The prevalence of different forms of malnutrition in terms of BMI was calculated for all of the 400 students who were categorized as thin, normal and overweight based on the NDHS reference. To assess the factors associated with undernutrition (thinness), the overweight students were excluded from the analysis. As the study included only females, there were no sex disaggregated analysis. The variations of the characteristics of those having normal nutrition and undernutrition were compared using a chi-square test. Subsequently, a multiple logistic regression analysis was used to assess the factors associated with undernutrition using a forward stepwise procedure. The factors included in the full model were the significant characteristics (*p* < 0.05) from the chi-square test results. The chances of multicollinearity of the independent variables identified as significant in the chi-square test were ruled out with reference to the variance inflation factor (VIF) and tolerance before performing the multiple logistic regression analysis. Crude and adjusted odd ratios and 95% confidence intervals for the associations were calculated. The quantitative data analysis was performed using Statistical Package for the Social Sciences (SPSS) version 16.

For the qualitative data analysis from interviews, the interview notes in Nepali were transcribed into English. The required information sorting, editing and revisions were made with the support of the interviewer and the principal investigator to ensure data integrity. Rigorous reading of the transcriptions was performed by principal investigator. As the dataset was small, a manual analysis using Microsoft word was performed rather than using other software. Altogether, 26 different codes were identified using description-focused coding. These codes were arranged alphabetically and then consolidated based on repetition of similar codes. All the codes were arranged in 5 different groups: nutrition status of adolescent girls in the district; reasons for poor nutritional status; programs for adolescent nutrition in the district; how well adolescent girls reached by the available programs; gaps in current policy and programs; and suggested recommendations to improve. This was later developed into 3 suitable themes: perspective on nutrition of adolescent girls, nutrition-focused program in the district and gap in present policies and programs and the way forward [46]. Themes were identified and categorized to detect findings that were prevalent (common across multiple key informants), insightful (unique to a few informants but a perspective that was important) or illustrative (in-depth comments that particularly described a concept or idea). At the end, the investigator ensured each theme and the content within them was meaningful and contributed to addressing the objective of the study.

For document review, policy, guidelines, strategies and other relevant reports were listed and read multiple times to identify key information such as the available interventions/current efforts; gaps to target adolescent nutrition and what can be done to improve. Notes were prepared that were later presented in the result and discussion section.

## 3. Results

**Figure 1:**
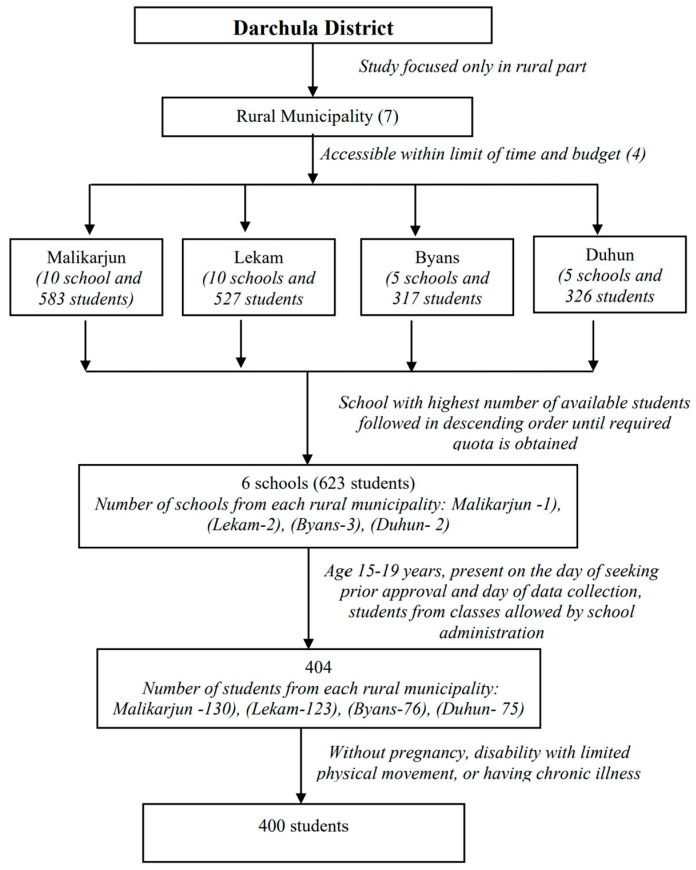
Selection of students for data collection.

In the four selected rural municipalities there were 30 secondary (grade 10 schools) and higher secondary schools (grade 12). According to data from the EDCU for the district, there were 1753 students in classes 9–12. As shown in Figure 1, of these, 33%, 30%, 19% and 18% were from Malikaarjun, Lekam, Duhun and Byans rural municipalities, respectively. The same proportion was used to select from 400 students, 130 (33%), 121 (30%), 75 (19%) and 74 (18%), respectively, from Malikaarjun, Lekam, Duhun and Byans rural municipalities. Based on their high numbers of student enrolment, one school from each of Malikarjun, Lekam and Duhun were selected, whereas three schools from Byans were included in the study because of the low numbers of the student populations. In the six selected schools, 623 students were enrolled in classes 9–12, of which 404 were aged 15–19 years. They were identified from classes approved by the administration for data collection. Among those 404, two students were pregnant and two had chronic illness and were hence excluded from the interview. Thus, the remaining 400 students were included in the study.

### 3.1. Prevalence of Nutritional Status of Students

Weights (kg) and heights (metres) of the 400 students were measured and their BMIs were calculated based on their weights and heights to identify their nutritional status. Their BMIs were classified into three categories: <18.5 kg/m^2^ as thin, 18.5–24.9 kg/m^2^ as normal and 25 and above as overweight. As shown in Table 1, of the 400 students, 284 (71%) had normal BMI, 99 (24.7%) were thin and 17 (4.3%) were overweight.

### 3.2. Characteristics of Thin and Normal Students

After excluding the 17 overweight students, a total of 383 students (284 normal BMI and 99 low BMI (thin)) were analysed for factors associated with nutritional status. The characteristics of thin and normal students were compared using the chi-square test. The results are presented separately for the different groups of the associated factors, including: socioeconomic characteristics—dietary intake, food availability and food-related practices; health, disease, mensuration and related practices; water, sanitation and hygiene at home; and knowledge and attitude related to nutrition.

Table 2 presents the chi-square test results for sociodemographic information of the thin and normal BMI students. Of all the factors, age of the students and fathers’ main occupations were significantly different (*p* < 0.05).

Table 3 shows the chi-square results for dietary intake, food availability and food-related practices among thin and normal students. The result shows significant differences for household food insecurity prevalence, how often food is purchased and kinds of food mostly bought.

Another group of variables were health and disease in terms of worm infestation, malaria, deworming and status of menstrual cycle along with food-related practices during mensuration and illness of thin and normal BMI students. In this group of variables, as shown in Table 4, only initiation of mensuration cycle had a significant difference.

Table 5 shows variables related to water, sanitation and hygiene at home among thin and normal BMI students. The practice of water treatment was the only significant factor.

As shown in Table 6, the chi-square test result of all the variables related to knowledge and attitude regarding nutrition- and food-related practices of thin and normal BMI students were insignificant.

### 3.3. Factors Associated with Thinness

Table 7 illustrates the crude analysis of similar significant factors to that presented in Table 2, Table 3, Table 4 and Table 5. After adjusting for other factors in the model, only a few factors remained significantly associated with thinness, i.e., the main occupation of the student’s father, particularly for those whose fathers have a monthly paying job (OR = 4.384: 95% CI = 1.135–16.928) and being employed in foreign countries (OR = 6.961; 95%CI = 1.649–29.377) and household food insecurity (OR = 2.079; 95% CI = 1.182–3.658). Additionally, grains/cereals/tuber is mostly bought for family food (OR = 9.487; 95% CI = 1.182–6.138) and the early initiation of mensuration cycle is significant (OR = 5.015; 95% CI = 1.257–20.011).

### 3.4. Emerging Themes Identified from Interview

Twelve eligible key informants were approached and nine agreed to participate in the key informant interview. Principals from three schools, health focal persons from two schools, chairpersons from three different wards and one health focal person from a ward office where the schools were located participated in the study. All the informants were male. Ward chairpersons had worked in their positions for the last four years whereas six other participants had more than fifteen years of experience of teaching at schools. Seven of the interviewees had obtained bachelor’s degree or above and the rest (two) had higher secondary education.

The findings of the interviews were organized in three different themes as follows:

#### 3.4.1. Perspective on Nutrition of Female Adolescents

All the key informants described the nutritional statuses of female adolescents in schools from average to good. At the same time, the interviewees also pointed out some nutritional related challenges such as: unsatisfactory growth as per age, not realizing nutrition-related issues even if they exist, lack of adequate and diversified diet, low awareness of families of the need for good adolescent nutrition, scarce food resources, insufficient knowledge and skills to use locally available nutritious food items. The chairperson of a ward mentioned, “There is lack of proper dietary practices. Local but nutritious food is ignored. Rather, the consumption of junk food has increased”. Some others also identified some socio-cultural factors such as food restriction during mensuration, gender discrimination, level of knowledge and economic status as the reasons for the nutrition-related issues.

#### 3.4.2. Adolescent-Nutrition-Focused Programs in the District

Almost all the key informants provided a list of similar nutrition-related programs from governmental and non-governmental sectors for school going adolescents in the district. Iron folic acid (IFA) distribution, deworming, health and nutrition education and pad making training were the most common ones. Besides this, the informants also shared their knowledge about some other infrequent and low scale but important activities such as food preparation skill demonstrations, nutrition assessment, awareness of prevention of child marriage, hygiene kit distribution, COVID-19 vaccination, etc. One ward chairperson emphasized, “Adolescents have benefited from the existing programs as a result there has been an improvement in sanitation and hygiene amongst them. IFA distribution has also helped to reduce anaemia among adolescents. There has been improvement in dietary practices as well”. The interventions enlisted by the informants were mostly aligned with the national school health and nutrition policy.

#### 3.4.3. Gap in the Present Policies and Programs and the Way Forward

Most of the participants felt there were enough nutrition-related programs for adolescents in the district, whereas a few expressed the need for more interventions. Most of the participants described issues at the implementation and management levels including delays in the release of budget, insufficient budget, delay in implementation, frequent discontinuation of activities, duplication, gaps in coordination among local stakeholders, etc. In addition to this, some also mentioned the issue of a top-to-bottom approach of policies and programs as a challenge. Participants cited examples of how programs, interventions and activities are implemented differently in other rural settings and suggested similar approaches be adopted in the district. A school principal added, “Programs are mostly planned to suite developed cities, rather programs need to be planned based on the overall scenario of the place for where they are meant to be”. Regarding increasing the effectiveness of existing programs and the required improvement in future, the need for understanding local context in the formulation of programs and coordination with local actors were suggested by most. A school health focal person expressed the need to expand the boundary of awareness programs and mentioned, “along with students, their guardians should also be involved in awareness activities to achieve better nutritional outcomes”. A school principals’ main focus was on the need to improve food-related practices and stated, “It is required to restrict the availability of junk or processed food from school canteen”. There were many other ideas on the way forward, a few of which were already in the policies and only require effective implementation. Provision of a school health nurse, availability of information, education, and communication (IEC) materials at school, including school management and guardians for making them aware of adolescent nutrition and their roles in it, new and innovative ideas, mid-day meals for all the students, separate curriculum for nutrition, proper budgeting and long-term programs were identified as critical for the improvement of the nutritional status of female adolescents in schools.

### 3.5. Nepal’s Commitment and Efforts around Adolescent Nutrition

Globally and nationally, there are various attempts aiming to end malnutrition by 2030. The adoption of Agenda 2030 and the commitment to the attainment of Sustainable Development Goals (SDGs) and the scaling up nutrition (SUN) movement launched in 2010 are a few key steps towards improved nutrition [38,47]. A review on nutrition policies, interventions and the attempt of countries committed to SUN in the context of adolescent nutrition calls for specific, evidence-based, multi-sectorial and innovative ideas to address the problem. This review and WHO guidelines for effective program implementation for adolescents identified research gaps and recommended for standard and effective scientific studies, regular and well-established monitoring and evaluation of the situation of adolescent nutrition, identification of gaps and evidence-based improvement on adolescent-nutrition-related topics [38,39]. Nepal has also committed to different nutrition-related global commitments such as the SDG and SUN and at the national level it has nutrition strategy 2077 (B.S), fifteen periodic plans (2019/20 to 2023/24), the multi-sector nutrition plan (MSNP), national health policies, Nepal health sector strategy plan (NHSSP) and the national agriculture development strategy (ADS) aiming to improve the nutritional status of pregnant and lactating women along with their children and adolescents. The Family Welfare Division (FWD), Department of Health Services (DOHS) and Ministry of Health and Population (MoHP), along with different supporting international and national organizations, are implementing nutrition-related interventions. Adolescent-girl-focused nutritional interventions in Nepal are mostly delivered through school health and nutrition programs [48,49]. Moreover, there are other inter-related interventions implemented from different sectors to contribute adolescent nutrition.

## 4. Discussion

This study aimed to identify the magnitude of malnutrition in form of BMI. The results show a 24.7% prevalence of undernutrition (thinness) among female students aged 15–19 years from the rural part of Darchula District. Different studies have reported variations in the prevalence of thinness among adolescent girls than found in this study. The percentage of thinness in this study is slightly lower than the finding by NDHS and a study conducted in 13 districts of Nepal, where 30% of late adolescent girls (15–19 years) and 42% of adolescent girls (10–19 years) were of low BMI [18,32]. A few possible reasons for the differences in the prevalence of thinness in this study are possible; it might be due to the sample size, study population, area of study, methodologies of the study, differences in the tools and existence of other factors among the students.

Binary logistic regression was used to identify factors associated with undernutrition in terms of thinness. The study demonstrated the significance associations between thinness and factors such as the main occupation of students’ father, HH food insecurity access prevalence, kind of food mostly bought by the family and initiation of the mensuration cycle. The association between father having monthly paying job or foreign employment as an occupation and thinness was found to be significant. Both types of occupation were identified to contribute to the high odds of having a low BMI. There are number of studies that have examined the relationship between occupation of father and BMI, but there are no similar findings. A study conducted among adolescents in a town in Ethiopia did not find any kind of association between the father’s occupation and the nutritional status of students [50]. However, a study based on secondary data analysis of the Indian Human Development Survey of 2011–2012 showed participants (adolescents aged 10–15 years) having migrant or migrant returnee fathers had lower BMI compared with the children of non-migrants [51]. On the other hand, a systematic review and meta-analysis did not find any association between migration of parents and the thinness of children and adolescents [52]. To understand the association of adolescent nutrition and the parent’s occupation, there is the need to appreciate other circumstances relating to the father’s job and foreign employment. For example, the income from a particular job or foreign employment as a low-paying job might compromise their ability to have the resources for good nutrition; whether a job or foreign employment limits a father’s availability to work on their own land and grow enough and nutritious food for making their family food secure; or how the absence of a father challenges the mother or HH head to make health and nutrition-related decisions or to fulfil the nutritional needs of the family. Therefore, an in-depth analysis is required to understand the pathways of how the father’s occupation impacts the nutrition of adolescents.

Again, a positive significant association between thinness and HH food insecurity prevalence was found in this study. There are studies that have found a significant level of association among HH food insecurity and the undernutrition of adolescents. A significant association between food insecurity and underweightness was found in a study conducted in urban slums in India among 418 teenage girls [53]. A significant association between HH food insecurity and thinness was reported by a systematic review and meta-analysis of 22 studies in Ethiopia among 17,854 adolescents [54]. Another systematic review showed a mixed result; around 44% to 50% of the literature included in the review found adolescents from food-insecure HHs thinner compared with their compatriots from food-secure HHs [55]. Food security at the HH level helps families to have diversified, balanced, adequate and appropriate food. At the same time, food insecurity leaves little or no choice about having nutritious food even though individuals may possess good knowledge and a proper attitude towards ideal recommended dietary practices but be undernourished.

Additionally, this study identified a significant association between the kind of food mostly bought and thinness. Adolescents from families mostly purchasing grains/tubers/cereals were at an increased risk of thinness. Though most grown food in the mountainous and hilly regions of Nepal are different types of grains, the staples are not sufficient for the whole year; hence, these regions face chronic food deficits for certain periods of the year [56]. These seasonal shortages result in the need to purchase more foodstuffs. Additionally, energy dense and less costly foodstuffs such as cereals remain the obvious choice for meals for most families in meeting their nutritional requirements in rural areas. There are studies that have investigated the association between the purchase of junk food and overnutrition, type of food available in the proximity and nutritional status and socio-economic status (SES) and type of food purchased. However, studies or reports related to the association between the kind of food purchased and thinness were limited. Understanding how exactly the type of food mostly purchased impacts BMI needs to be further studied to possibly explore the role of amount of intake of grain, calorie demands for the body’s physical functional activities, the average expenditure for different food items and any other intertwining factors.

Lastly, initiation of mensuration cycle was also found to be statistically significant in the current study. Although this study has fewer students whose menarche had not occurred at the time of data collection, the analysis shows that females yet to start their mensuration cycle are more likely to be thin. A study conducted in the rural communities of different districts of Ethiopia also revealed a similar result, where pre-mensuration girls were more likely to be thin than those who had already had their menarche. This is also supported by two other studies, one conducted in urban and rural parts of China and another a community-based cross-sectional study conducted in an urban settlement in Delhi among adolescent girls [20,21]. Both studies noted girls who had begun their mensuration cycle had a higher BMI than their counterparts [20,21]. The observed association in most of the studies could be explained by considering the physiological changes in the body and attaining a spurt in growth with advancing age, especially after menarche during adolescence [57].

The study aimed to identify gaps in current efforts and present recommendations for the improvement of adolescent nutrition through KII and review of policy and interventions. The findings through KII have highlighted the existence of problems with direct or indirect relationships with undernutrition such as the lack of tracking of nutritional status, deficit in proper diet, unsatisfactory awareness of adolescent nutrition, poor economic status and gender inequalities. The review of the country’s current commitments and efforts on nutrition, with focus on school health and nutrition programs, has given a picture of what is being attempted, the gaps and what needs to be done for improvement. As contained in the WHO guideline “implementing effective action for improving adolescent nutrition”, there are areas to improve in existing interventions to address adolescent malnutrition. Though awareness programs exist, minimal work has been conducted exploring and covering new but important nutrition-related topics such as promotion of healthy diets, lifestyle modification for a healthy life and various determinants of nutrition. WHO guidelines recommend to address prominent nutritional issues due to insufficient intake of the recommended daily allowance of nutrients; there is limited or no access to vitamin A supplementation, use of micronutrient powder for instant fortification and fortified food for adolescents [48]. Through MSNP, school health and nutrition programs and with the support of various agencies, adolescents are approached from various interlinked sectors to improve the nutritional status of this group. Yet, there is much more work to do in broadening the scope of the multi-sectorial approach to incorporate adolescents’ nutrition. Child club, the concept of adolescent friendly services in school and health care settings are also in place but are not prominent enough to reflect this developmental stage and adolescents’ unique needs in order to strongly advocate for adolescent-targeted interventions. Furthermore, the One School One Nurse Program of the government for creating awareness and sensitising adolescents towards the various aspects related to health and nutrition, is a much-touted program that has already started but is yet to be scaled up [49]. In addition to the gaps in existing interventions, this study, which is consistent with findings of various other reviews, guidelines and consultation meetings, identified shortcomings in areas pertaining to: the assessment of the problem of undernutrition focused on adolescents and its determinants; the limited understanding of the diverse needs of obtaining the optimal levels of health and nutrition for female adolescents considering the prevailing physical, mental, social, economic, political and environmental contexts; the program effectiveness assessment studies; and the studies to bring innovation in approaches to understand and address the problem of undernutrition of adolescents.

The study provides recommendations to stakeholders for improvement in policies and programs to best address the nutritional needs of school-going female adolescents. The prevalence of malnutrition in this study demonstrates the need for regular nutritional assessment and timely management of malnutrition. These kinds of assessments can be a part of a regular growth monitoring from health centres and schools and could have this as its scheduled program. As the study identified significant associations with different individual, social and food-related factors with thinness, schools need to have more organized and effective interventions to improve the knowledge of adolescents regarding various determinants of nutrition. Additionally, the findings enjoin stakeholders to strengthen a multi-sectorial approach for addressing the diverse nutritional needs of this unique group. Along with specific interventions, nutrition-sensitive interventions from various sectors will be equally important to improve the various social and food-related factors. Based on KII interviews and the review of school health and nutrition programs, it is recommended that governments and school management urgently scale-up programs to support activities related to the promotion, prevention and management of adolescent health and nutrition at school levels. Furthermore, evidence-based policy formulation, its contextual modifications relevant to the targeted population and innovations in interventions are highly recommended. Along with programmatic recommendations, the findings of this study have identified further areas to research. Studies to understand the magnitude of the problem of undernutrition in this group from different settings and its determining factors is highly recommended. As the problem and its cause varies with changes in the context, it is important to design studies to understand what works and what does not, which will in future support the formulation of effective policies and programs. Systematic surveillance, monitoring and evaluation studies of current interventions are needed for timely identification of gaps and corrective actions and to bring innovations to conventional programmatic approaches.

This study is limited by the use of quota sampling (non-probability) as it limits the generalization of its findings. This was due to the unexpected challenges of the COVID-19 pandemic. Therefore, studies using a probability sampling design are recommended for future research.

## 5. Conclusions

The study has revealed that about one-third of the late adolescent female students in the rural municipalities of Darchula District were suffering from malnutrition in form of low BMI. The study also found that the father’s main occupation, family food insecurity, having grain/root/tuber food as the family’s main food, and delayed initiation of menarche increased the odds of thinness among female adolescents. Most of the interventions implemented in the district are aligned with the national school health nutrition programs but need improvements. The findings the study further highlights gaps in the existing efforts at the programmatic level along with a huge knowledge gap in undernutrition and related factors among school-going female adolescents. Formulation of targeted policies and programs and their effective implementation are essential.

## Figures and Tables

**Table 1:** BMI status of students.

BMI Status	Frequency (*n* = 400)	Percentage
Thin	99	24.7
Normal	284	71
Overweight	17	4.3
Total	400	100

BMI = Body Mass Index.

**Table 2:** Socio-demographic factors of thin and normal BMI students.

Factors	Thin (*n* = 99)	Normal BMI (*n* = 284)	*p* Value
Age of students (in completed years)			
15–16	78 (78.8)	187 (65.8)	
17–19	21 (21.2)	97 (34.2)	0.016
Religion			
Hindu	98 (99)	276 (97.2)	0.307
Christian	1 (1)	8 (2.8)	
Ethnicity			
Dalit	4 (4)	13 (4.6)	0.581
Brahmin/Chettri	90 (90.9)	248 (87.3)	
Other	5 (5.1)	23 (8.1)	
Size of the family			
<5 members	13 (13.1)	42 (14.8)	0.686
≥5 members	86 (86.9)	242 (85.2)	
Head of the household			
Father	63 (63.6)	185 (65.1)	0.81
Mother	12 (12.1)	25 (8.8)	
Grandfather	14 (14.1)	42 (14.8)	
Grandmother	10 (10.1)	30 (10.6)	
Education of student’s father			
Illiterate	9 (9.1)	24 (8.5)	0.816
Informal education	6 (6.1)	22 (7.7)	
Less than primary	6 (6.1)	19 (6.7)	
Primary level	14 (14.1)	26 (9.2)	
Lower secondary or Secondary	41 (41.4)	124 (43.7)	
Higher secondary and above	23 (23.2)	69 (24.3)	
Education of student’s mother			
Illiterate	34 (34.3)	107 (37.7)	0.551
Informal education	26 (26.3)	69 (24.3)	
Less than primary	5 (5.1)	25 (8.8)	
Primary level	8 (8.1)	29 (10.2)	
Lower secondary or Secondary	16 (16.2)	31 (10.9)	
Higher secondary and above	10 (10.1)	23 (8.1)	
Main occupation of student’s father			
Monthly paying job (GO/NGO/Private)	23 (23.2)	53 (18.7)	0.005
Agriculture	53 (53.5)	142 (50)	
Business	5 (5.1)	41 (14.4)	
Foreign employee	15 (15.2)	21 (7.4)	
Others (daily wage, contractor, etc)	3 (3)	27 (9.5)	
Main occupation of student’s mother			
Housewife	35 (35.4)	113 (39.8)	0.505
Agriculture	60 (60.6)	151 (53.2)	
Monthly paying job (GO/NGO/Private)	3 (3)	17 (6)	
Other (daily wage, contractor, etc)	1 (1)	3 (1.1)	
Monthly income of family			
Up to NRs. 2000	26 (26.3)	83 (29.2)	0.792
NRs.2001 to 5000	27 (27.3)	79 (27.8)	
NRs.5001 to 10,000	17 (17.2)	53 (18.7)	
>NRs.10,000	29 (25.8)	69 (24.3)	

GO = Government Organization, NGO = Non-Government Organization.

**Table 3:** Dietary intake, food availability and food-related practice factors for thin and normal BMI students.

Factors	Thin (*n* = 99)	Normal BMI (*n* = 284)	*p* Value
Diversity on 24 h recall			
<5 food groups	25 (25.3)	49 (17.3)	0.083
≥5 food groups	74 (74.7)	235 (82.7)	
Household food insecurity assessment			
Food Secure	60 (60.6)	217 (76.4)	0.002
Food insecure	39 (39.4)	67 (23.6)	
How often do you buy food			
Daily	14 (14.1)	62 (21.8)	0.001
Weekly	11 (11.1)	71 (25)	
Monthly	17 (17.2)	46 (16.2)	
Randomly	57 (57.6)	105 (37)	
Kind of food mostly bought in the family			
Grains/roots/tubers	51 (51.5)	81 (28.5)	0.002
Pulses/cereals/legume	12 (12.1)	50 (17.6)	
Meat and meat products	14 (14.1)	58 (20.4)	
Vegetables	13 (13.1)	48 (16.9)	
Fruits	8 (8.1)	30 (10.6)	
Other (egg and dairy products)	1 (1)	17 (6)	
Priority during serving of nutritious food			
All served equally	80 (80.8)	212 (74.6)	0.382
Children	5 (5.1)	31 (10.9)	
Pregnant, lactating	8 (8.1)	24 (8.5)	
Other (Male, elderly)	6 (6.1)	17 (6)	
Meals consumed per day			
Once a day	0	2 (0.7)	0.244
Twice a day	5 (5.1)	32 (11.3)	
Thrice a day	83 (83.8)	217 (76.4)	
≥Four times a day	11 (11.1)	33 (11.6)	
Are you vegetarian or non-vegetarian			
Vegetarian	16 (16.2)	57 (20.1)	0.394
Non-Vegetarian	83 (83.8)	227 (79.9)	
Consume junk or processed food			
Yes	92 (92.9)	251 (88.4)	0.203
No	7 (7.1)	33 (11.6)	
Missing of any meal(s) of a day			
No	39 (39.4)	114 (40.1)	0.896
Yes	60 (60.6)	170 (59.9)	

**Table 4:** Disease and mensuration and related practices for thin and normal BMI students.

Factors	Thin (*n* = 99)	Normal BMI (*n* = 284)	*p* Value
Initiation of mensuration cycle			
Yes	93 (93.9)	280 (98.6)	0.012
No	6 (6.1)	4 (1.4)	
Food restriction during mensuration (*n* = 373)			
Yes	62 (66.7)	177 (63.2))	0.548
No	31 (33.3)	103 (36.8)	
Ever suffered from worm infestation			
Yes	34 (34.3)	130 (45.8)	0.107
No	59 (59.6)	144 (50.7)	
Don’t know	6 (6.1)	10 (3.5)	
Taken anthelminthic drug in last 6 months			
Yes	77 (77.8)	238 (83.8)	0.094
No	17 (17.2)	42 (14.8)	
Don’t know	5 (5.1)	4 (1.4)	
Type of food you eat during illness			
More nutritious than usual	62 (62.6)	176 (62)	0.090
Rice & dal only	28 (28.3)	56 (19.7)	
Normal family food	9 (9.1)	51 (18)	
Frequency of eating during illness			
As usual	13 (13.1)	46 (16.2)	0.171
More than usual	4 (4)	28 (9.9)	
Less than usual	82 (82.8)	208 (73.2)	

**Table 5:** Water, sanitation and hygiene at home factors for thin and normal BMI students.

Factors	Thin (*n* = 99)	Normal BMI (*n* = 284)	*p* Value
Main source of drinking water			
Tap water	64 (64.6)	184 (64.8)	0.189
Public tap	31 (31.3)	71 (25)	
Well	3 (3)	15 (5.3)	
Other	1 (1)	14 (4.9)	
Is water treated			
Yes	42 (42.4)	156 (54.9)	0.028
No	57 (57.6)	123 (43.3)	
Don’t know	0	5 (1.8)	
What do you use to wash your hands			
Water only	4 (4)	5 (1.8)	0.23
Water and soap	93 (93.9)	277 (97.5)	
Water and Ash/mud	2 (2)	2 (0.7)	
Fixed latrine for HH available			
Yes	94 (94.9)	274 (96.5)	0.499
No	5 (5.1)	10 (3.5)	
Always wear shoes when going outside			
Yes	96 (97)	266 (93.7)	0.213
No	3 (3)	18 (6.3)	

**Table 6:** Knowledge and attitude related to nutrition-related factors for thin and normal BMI students.

Factors	Thin (*n* = 99)	Normal BMI (*n* = 284)	*p* Value
Level of knowledge			
Average	17 (17.2)	43 (15.1)	0.7
Satisfactory	72 (72.7)	213 (75)	
Good	10 (10.1)	25 (8.8	
Attitude towards health or nutrition problems			
Likely	64 (64.6)	172 (60.6)	0.745
Neutral	24 (24.2)	74 (26.1)	
Unlikely	11 (11.1)	38 (13.4)	
Perceived benefits or ideal or recommended nutrition-related practices			
Positive	90 (90.9)	257 (90.5)	0.765
Uncertain	8 (8.1)	21 (7.4)	
Negative response	1 (1)	6 (2.1)	
Perceived barriers regarding the difficulties arising if engaging in ideal nutrition			
Likely	90 (90.9)	244 (85.9)	0.203
Not Sure	8 (8.1)	26 (9.2)	
Unlikely	1 (1)	14 (4.9)	
Self-confidence to adopt the desired practice			
Positive	74 (74.7)	184 (64.8)	0.188
Uncertain	19 (19.2)	74 (26.1)	
Negative	6 (6.1)	26 (9.2)	

**Table 7:** Factors associated with low BMI (thinness).

Factors	Crude Odds Ratio (OR) (95% Confidence Interval (CI))	*p*-Value	Adjusted Odds Ratio (AOR) (95% CI)	*p*-Values
Age of the students in completed years				
15–16 years	1			
17–19 years	0.519 (0.302–0.891)	0.017	NS	
Main occupation of respondent’s father				
Other	1		1	
Monthly paying job (GO/NGO/Private)	3.906 (1.076–14.178)	0.038	4.384 (1.135–16.928)	0.032
Agriculture	3.359 (0.978–11.536)	0.054	3.114 (0.865–11.213)	0.082
Business	1.098 (0.242–4.976)	0.904	1.117 (0.232–5.372)	0.89
Foreign employment	6.429 (1.643- 25.159)	0.008	6.961(1.649–29.377)	0.008
HH food insecurity access prevalence				
Food secure	1		1	.
Food insecure	2.105 (1.293–3.427)	0.003	2.079 (1.182–3.658)	0.011
Kind of food mostly bought by the family				
Other (dairy products, eggs)	1		1	.
Grains/roots/tubers	10.704 (1.382–82.896)	0.023	9.487 (1.182–76.138)	0.034
Pulses/cereals/legumes	4.080 (0.493–33.751)	0.192	4.731 (0.544–41.108)	0.159
Vegetables and green vegetables	4.103 (0.503–33.494)	0.188	3.539 (0.416–30.134)	0.248
Meat and meat products	4.604 (0.559–37.894)	0.156	4.419 (0.518–37.667)	0.174
Fruits	4.533 (0.522–39.401)	0.171	4.478 (0.484–41.465)	0.187
How often you buy food				
Daily	1			
Weekly	0.416 (0.214–0.808)	0.01	NS	
Monthly	0.285 (0.140–0.582)	0.001		
Randomly	0.681 (0.358–1.295)	0.241		
Initiation of mensuration cycle				
Yes	1		1	.
No	4.516 (1.247–16.352)	0.022	5.015 (1.257–20.011)	0.022
Water treatment to make it safer to drink				
No	1			
Yes	0.581 (0.366–0.923)	0.022	NS	
Don’t know	0	0.999		

Note: NS—Not significant (*p* > 0.05) adjusted odds ratio.

## Data Availability

Not applicable.

## References

[1] World Health Organization (2018). Malnutrition [Fact Sheet]. Details. https://www.who.int/news-room/fact-sheets/detail/malnutrition.

[2] Benedict R.K., Schmale A., Namaste S. (2018). Adolescent Nutrition 2000–2017: DHS Data on Adolescents Age 15–19.

[3] World Health Organization, Regional Office for South-East Asia (2006). Adolescent Nutrition: A Review of the Situation in Selected South-East Asian Countries.

[4] Spear B.A. (2002). Adolescent Growth and Development. J. Am. Diet. Assoc..

[5] Savarino G., Corsello A., Corsello G. (2021). Macronutrient balance and micronutrient amounts through growth and development. Ital. J. Pediatr..

[6] United Nations Children’s Fund (2018). Child Marriage: Latest Trend and Future Prospects. https://data.unicef.org/wp-content/uploads/2018/07/Child-Marriage-Data-Brief.pdf.

[7] World Health Organization (2022). Adolescent Pregnancy [Fact Sheet]. https://www.who.int/news-room/fact-sheets/detail/adolescent-pregnancy.

[8] Khanal V., Sauer K., Karkee R., Zhao Y. (2014). Factors associated with small size at birth in Nepal: Further analysis of Nepal Demographic and Health Survey 2011. BMC Pregnancy Childbirth.

[9] Aguayo V.M., Paintal K. (2017). Nutrition in adolescent girls in South Asia. BMJ.

[10] Black R.E., Victora C.G., Walker S.P., Bhutta Z.A., Christian P., de Onis M., Ezzati M., Grantham-McGregor S., Katz J., Martorell R. (2013). Maternal and child undernutrition and overweight in low-income and middle-income countries. Lancet.

[11] Fiorentino M. (2015). Malnutrition in School-Aged Children and Adolescents in Senegal and Cambodia: Public Health Issues and Interventions. Ph.D. Dissertation.

[12] Anthony D. (2011). The State of the World’s Children 2011—Adolescence: An Age of Opportunity.

[13] Christian P., Smith E.R. (2018). Adolescent Undernutrition: Global Burden, Physiology, and Nutritional Risks. Ann. Nutr. Metab..

[14] Nithya D.J., Bhavani R.V. (2018). Dietary diversity and its relationship with nutritional status among adolescents and adults in rural India. J. Biosoc. Sci..

[15] Keats E.C., Rappaport A.I., Shah S., Oh C., Jain R., Bhutta Z.A. (2018). The Dietary Intake and Practices of Adolescent Girls in Low- and Middle-Income Countries: A Systematic Review. Nutrients.

[16] Radhika M.S., Swetha B., Kumar B.N., Krishna N.B., Laxmaiah A. (2018). Dietary and nondietary determinants of nutritional status among adolescent girls and adult women in India. Ann. N. Y. Acad. Sci..

[17] Leroy J.L., Ruel M., Sununtnasuk C., Ahmed A. (2018). Understanding the determinants of adolescent nutrition in Bangladesh. Ann. N. Y. Acad. Sci..

[18] Aryal K.K., Mehata K.R., Chalise B., Mehata S., Sapkota F., Dhimal M., Jha B.K., Karki K.B. (2016). Adolescent Nutrition Survey in Nepal, 2014.

[19] Yemaneh Y., Girma A., Niguse W., Hailu D., Alemayehu T., Mesfin F., Abera A., Dagnachew E. (2017). Undernutrition and its associated factors among adolescent girls in rural community of Aseko district, Eastern Arsi Zone, Oromia region, Eastern Ethiopia. World Health.

[20] Wang Z., Liu J., Shuai H., Cai Z., Fu X., Liu Y., Xiao X., Zhang W., Krabbendam E., Liu S. (2021). Mapping global prevalence of depression among postpartum women. Transl. Psychiatry.

[21] Acharya A., Reddaiah V.P., Baridalyne N. (2006). Nutritional status and menarche in adolescent girls in an urban resettlement colony of South Delhi. Indian J. Community Med..

[22] Bhandari S., Sayami J.T., Thapa P., Sayami M., Kandel B.P., Banjara M.R. (2016). Dietary intake patterns and nutritional status of women of reproductive age in Nepal: Findings from a health survey. Arch. Public Health.

[23] Shah G.S., Bhatta N.K., Singh R.R., Bhatta M., Kanodia P. (2016). A study of anemia among adolescent girls in eastern part of Nepal. J. Coll. Med. Sci.—Nepal.

[24] Branca F., Piwoz E., Schultink W., Sullivan L.M. (2015). Nutrition and health in women, children, and adolescent girls. BMJ.

[25] Johnson R.K., Lamb M., Anderson H., Pieters-Arroyo M., Anderson B.T., Bolanos G.A., Asturias E.J. (2019). The global school-based student health survey as a tool to guide adolescent health interventions in rural Guatemala. BMC Public Health.

[26] Kar A., Slavchevska V., Kaaria S., Taivalmaa S.L., Mane E., Ciacci R., Hoberg Y.T. (2018). Male Outmigration and Women’s Work and Empowerment in Agriculture: The Case of Nepal and Senegal.

[27] Madjdian D.S., Bras H.A. (2016). Family, gender, and women’s nutritional status: A comparison between two Himalayan communities in Nepal. Econ. Hist. Dev. Reg..

[28] Desbouys L., Méjean C., De Henauw S., Castetbon K. (2020). Socio-economic and cultural disparities in diet among adolescents and young adults: A systematic review. Public Health Nutr..

[29] Krolner R., Rasmussen M., Brug J., Klepp K.I., Wind M., Due P. (2011). Determinants of fruit and vegetable consumption among children and adolescents: A review of the literature. Part II: Qualitative studies. Int. J. Behav. Nutr. Phys. Act..

[30] Das J.K., Salam R.A., Thornburg K.L., Prentice A.M., Campisi S., Lassi Z.S., Koletzko B., Bhutta Z.A. (2017). Nutrition in adolescents: Physiology, metabolism, and nutritional needs. Ann. N. Y. Acad. Sci..

[31] Government of Nepal, National Planning Commission Secretariat (2012). National Population and Housing Census 2011 (National Report). https://unstats.un.org/unsd/demographic-social/census/documents/Nepal/Nepal-Census-2011-Vol1.pdf.

[32] Ministry of Health, New ERA/Nepal, ICF (2017). Nepal Demographic and Health Survey 2016. http://dhsprogram.com/pubs/pdf/FR336/FR336.pdf.

[33] Mahumud R.A., Sultana M., Sarker A.R. (2017). Distribution and Determinants of Low Birth Weight in Developing Countries. J. Prev. Med. Public Health.

[34] Bhandari S., Sayami J., Ricky Raj K.C., Banjara M. (2015). Prevalence of congenital defects including selected neural tube defects in Nepal: Results from a health survey. BMC Pediatr..

[35] Kc A., Wrammert J., Nelin V., Ewald U., Clark R., Malqvist M. (2015). Level of mortality risk for babies born preterm or with a small weight for gestation in a tertiary hospital of Nepal. BMC Public Health.

[36] United Nations Development Programme (UNDP) (2014). Nepal Human Development Report 2014. Beyond Geography: Unlocking Human Potential. http://www.hdr.undp.org/sites/default/files/nepal_nhdr_2014-final.pdf.

[37] United Nations Educational, Scientific and Cultural Organization (UNESCO) (2019). Education and Literacy. Participantion in Education. http://uis.unesco.org/country/np#slideoutmenu.

[38] Khara T., Mates E. (2015). Adolescent Nutrition. Policy and Programming in SUN+ Countries.

[39] World Health Organization (2018). Guideline: Implementing Effective Actions for Improving Adolescent Nutrition.

[40] Coates J., Spindale A., Bilinsky P. (2007). Household Food Insecurity Access Scale (HFIAS) for Measurement of Household Food Access: Indicator Guide.

[41] FAO, FHI 360 (2016). Minimum Dietary Diversity for Women: A Guide for Measurement.

[42] Marías Y.F., Glasauer P. (2014). Guidelines for Assessing Nutrition-Related Knowledge, Attitudes and Practices.

[43] Diaz A., Laufer M.R., Breech L.L. (2006). Menstruation in girls and adolescents: Using the menstrual cycle as a vital sign. Pediatrics.

[44] Nelima D. (2015). Prevalence and Determinants of Anaemia among Adolescent Girls in Secondary Schools in Yala Division Siaya District, Kenya. Univ. J. Food Nutr. Sci..

[45] Bundy D.A., de Silva N., Horton S., Patton G.C., Schultz L., Jamison D.T. (2017). Child and Adolescent Health and Development.

[46] Adu P. (2019). A Step-by-Step Guide to Qualitative Data Coding.

[47] Grosso G., Mateo A., Rangelov N., Buzeti T., Birt C. (2020). Nutrition in the context of the Sustainable Development Goals. Eur. J. Public Health.

[48] Department of Health Services, Ministry of Health and Population (2022). Annual Report (2020/21). https://dohs.gov.np/wp-content/uploads/2022/07/DoHS-Annual-Report-FY-2077-78-date-5-July-2022-2022_FINAL.pdf.

[49] Ministry of Health and Population, Ministry of Education (2020). Joint Action Plan (2071/72-2076/77): School Health and Nutrition. https://faolex.fao.org/docs/pdf/nep191267.pdf.

[50] Gebremariam H., Seid O., Assefa H. (2015). Assessment of nutritional status and associated factors among school going adolescents of Mekelle City, Northern Ethiopia. Int. J. Nutr. Food Sci..

[51] Lei L., Desai S., Chen F. (2020). Fathers’ migration and nutritional status of children in India: Do the effects vary by community context?. Demogr. Res..

[52] Fellmeth G., Rose-Clarke K., Zhao C., Busert L.K., Zheng Y., Massazza A., Sonmez H., Eder B., Blewitt A., Lertgrai W. (2018). Health impacts of parental migration on left-behind children and adolescents: A systematic review and meta-analysis. Lancet.

[53] Singh J.K., Acharya D., Rani D., Gautam S., Thapa Bajgain K., Bajgain B.B., Park J.H., Yoo S.J., Poder T.G., Lewin A. (2021). Underweight and Associated Factors Among Teenage Adolescent Girls in Resource-poor Settings: A Cross-sectional Study. Risk Manag. Healthc. Policy.

[54] Berhe K., Kidanemariam A., Gebremariam G., Gebremariam A. (2019). Prevalence and associated factors of adolescent undernutrition in Ethiopia: A systematic review and meta-analysis. BMC Nutr..

[55] Dewi N.U., Nurulfuadi N., Aiman U., Hartini D.A., Pradana F., Bohari B. (2020). Food Insecurity and Anthropometry in Adolescents: A Literature Review. Open Access Maced. J. Med. Sci..

[56] Paudel M.N. (2016). Prospects and limitations of agriculture industrialization in Nepal. Agron. J. Nepal.

[57] Abraham S., Boyd C., Lal M., Luscombe G., Taylor A. (2009). Time since menarche, weight gain and body image awareness among adolescent girls: Onset of eating disorders. J. Psychosom. Obstet. Gynaecol..

